# A 5-year Chinese longitudinal case report on the malignant transformation of spinal Paget disease

**DOI:** 10.1097/MD.0000000000047190

**Published:** 2026-01-30

**Authors:** Yu Wang, Linlin Zhang, Huilin Yang, Qin Zhang

**Affiliations:** a Department of Orthopaedics, The First Affiliated Hospital of Soochow University, Suzhou, Jiangsu, China.

**Keywords:** alkaline phosphatase, genetic predisposition, malignant transformation, osteoclast dysfunction, Paget disease of bone, spinal cord compression, SQSTM1 mutation, ZNF687 mutation

## Abstract

**Rationale:**

Paget disease of bone (PDB) is a metabolic disorder typically regarded as benign, yet it carries a <1% risk of malignant transformation into osteosarcoma malignant transformation into osteosarcoma, particularly with spinal involvement. Differentiation from other aggressive spinal lesions can be challenging. In Asian populations, prognostic data on Paget sarcoma are scarce. The significance of dual SQSTM1 and ZNF687 mutations and a rapid alkaline phosphatase (ALP) surge as indicators of malignant transformation remains to be fully elucidated.

**Patient concerns:**

We report a case of spinal Paget sarcoma in a patient with a 5-year history of PDB. The patient presented with progressive symptoms culminating in acute neurological deterioration, including bilateral lower limb weakness and bowel/bladder dysfunction, indicating severe spinal cord compromise.

**Diagnoses:**

The diagnosis of malignant transformation was based on the clinical course, serial imaging showing progressive spinal involvement, a sharp rise in ALP (a hydrolase enzyme that is predominantly found in the liver and bone, and elevated serum levels of which serve as a key biomarker for hepatobiliary disorders or abnormal bone metabolism) from >800 U/L to 3100 U/L, and genetic testing confirming dual SQSTM1 and ZNF687 mutations.

**Interventions:**

The patient underwent surgical intervention, targeted therapy, and other supportive treatments. An emergency workup was performed due to rapid neurological decline.

**Outcomes:**

Despite treatment, the patient experienced a rapid functional decline, with lower limb strength dropping to grade 1 by postoperative day 40, reflecting a poor prognosis associated with advanced Paget sarcoma. During the 2025 follow-up, the family was approached but declined to disclose the patient’s status. The patient’s survival status remains unknown as of the last follow-up.

**Lessons:**

In patients with PDB and spinal involvement, a rapid increase in ALP, especially in the context of specific genetic mutations like SQSTM1 and ZNF687, may be a critical indicator of malignancy. This case highlights the need for heightened clinical vigilance, enhanced genetic monitoring, and consideration of early biopsy to enable timely intervention and improve patient outcomes in this rare but devastating complication.

## 1. Introduction

Paget disease of bone (PDB), also referred to as osteitis deformans, is a metabolic bone disorder characterized by the abnormal function of osteoclasts, typically presenting as a benign condition.^[1,2]^ Paget disease of the bone is the second most common metabolic bone disease in the world, with a prevalence of 1.5 to 8.3%.^[3]^ However, Paget disease has been reported with notably low prevalence across East Asian countries, such as China, Japan, and Korea.^[4]^ The probability of malignancy in Paget disease is <1%, and the most common skeletal sites are, in descending order: femur, pelvis, humerus, and skull, with the spine rarely involved. The presence of Paget disease at an unconventional site is often a sign that there is a greater chance of malignancy.^[5]^

In this case report, we present a Chinese patient with PDB who was followed over a 5-year period and exhibited both classic humeral involvement and abnormal bone metabolism across multiple vertebrae. The case is notably characterized by its rare spinal manifestation and eventual malignant transformation to osteosarcoma, which was confirmed via intraoperative vertebral biopsy. Accompanying this progression was a rapid onset of symptoms, including incomplete paralysis. Genetic analysis revealed a rare double mutation in both SQSTM1 and ZNF687, highlighting a potential genetic predisposition for severe and atypical disease phenotypes. To our knowledge, this represents one of the few documented cases of malignant vertebral osteosarcoma complicating PDB within an Asian individual. Furthermore, this report offers comprehensive longitudinal data – including serial imaging, bone metabolism indices, biochemical profiles, and genetic testing – that provide valuable insights for diagnosing and treating Paget disease, particularly in underrepresented populations.

## 2. Case report

### 2.1. Patient information

In June 2019, a 55-year-old woman presented with low back pain and was diagnosed with Paget disease based on radiographic findings of multifocal osteolytic lesions, cortical hyperplasia, osteosclerosis, and elevated alkaline phosphatase (ALP > 800 U/L). The patient was initiated on bisphosphonate therapy with annual follow-up. On November 14, 2024, she developed acute neurological symptoms, including rapidly ascending numbness (reaching the chest level within 24 hours), lower limb weakness (right grade 4, left grade 3), and urinary and bowel dysfunction. Physical examination revealed sensory deficits below the sternal angle and impaired motor function.

### 2.2. Diagnostic evaluation

**Figure 1.**
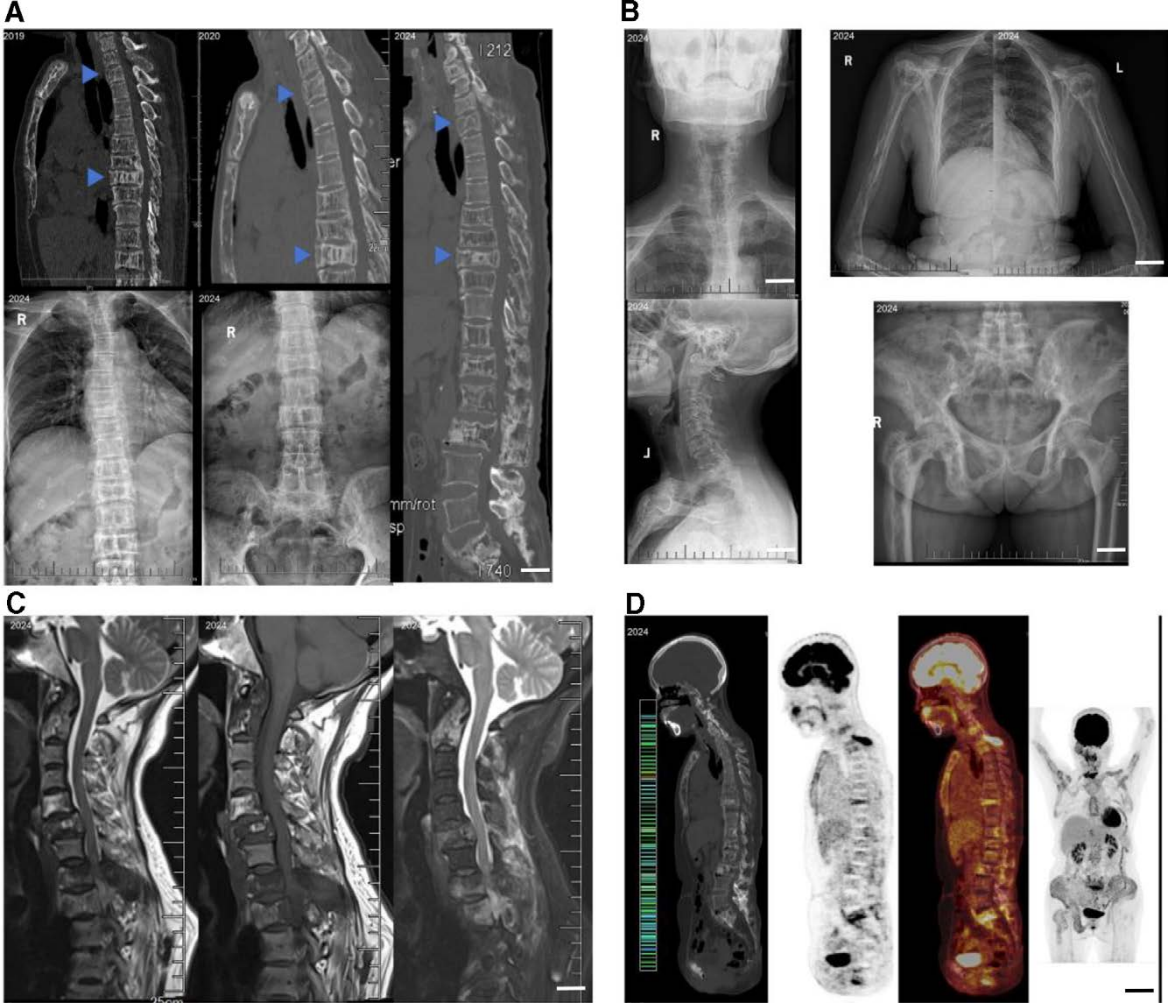
Images data across different years. (A) Thoracic CT images from different years and the most recent thoracolumbar X-ray. The blue arrows indicate vertebrae with progressive bone destruction and decreasing vertebral height (scale bar = 4 cm); (B) The most recent X-rays of the cervical spine, humerus, pelvis, and femoral neck (scale bar = 3 cm); (C) MRI of the cervical and part of the thoracic spine, showing abnormal signals in the vertebral bodies and their appendages, as well as in the surrounding soft tissues (scale bar = 2 cm); (D) PET-CT imaging of the patient’s spine (scale bar = 10 cm). CT = computed tomography, MRI = magnetic resonance imaging, PET-CT = positron emission tomography-computed tomography.

By October 2024, her ALP level had risen sharply to 3100 U/L, indicating disease progression. A detailed examination of the patient was conducted, encompassing X-rays and magnetic resonance imaging of the cervicothoracic spine, bilateral humerus, shoulder and hip (2024). The analysis revealed multiple areas of low-density bone destruction in the cervical, thoracic and lumbar spine. No calcification of the anterior longitudinal ligament was observed. Multiple low-density bone disruptions were identified in the left clavicle, bilateral proximal humeri, bilateral scapulae, pelvis and right proximal femur. Multiple bony disruptions were observed in the craniofacial skeleton, including some of the ribs on both sides, the left clavicle, bilateral scapulae, sternum, multiple vertebrae and appendages, and the pelvis, which was locally enlarged and partially showed a ground-glass density shadow. Computed tomography scans and reconstructions of the cervicothoracolumbar spine (June 18, 2019 and November 15, 2024). The analysis revealed compression, flattening, and posterior protrusion of the C6, T9, and L3 vertebral bodies, and increased narrowing of the spinal canal when compared to the 2019 images. The study also identified slight posterior displacement of the L3 vertebral body and flattening of multiple vertebral bodies in the cervical, thoracic, lumbar, and sacral spine. The margins of the vertebral bodies were osteophytic and spiky. L3-5 intervertebral discs were protruding, with posterior compression of the dural sac. Cervicothoracic magnetic resonance imaging T1 and T2, sagittal + coronal. Multiple vertebral wedge-shaped changes and bone destruction can be observed in the vertebral bodies and appendages. Multiple intraosseous patches of mixed high and low signals are present throughout the body. The vertebral bodies and attachments exhibited varying degrees of osteophytic changes, with no discernible narrowing of the intervertebral space. Herniated discs were identified at C3-7 and L3-5. Positron emission tomography-computed tomography demonstrated increased glucose metabolism in multiple skeletal sites (Fig. 1).

**Figure 2.**
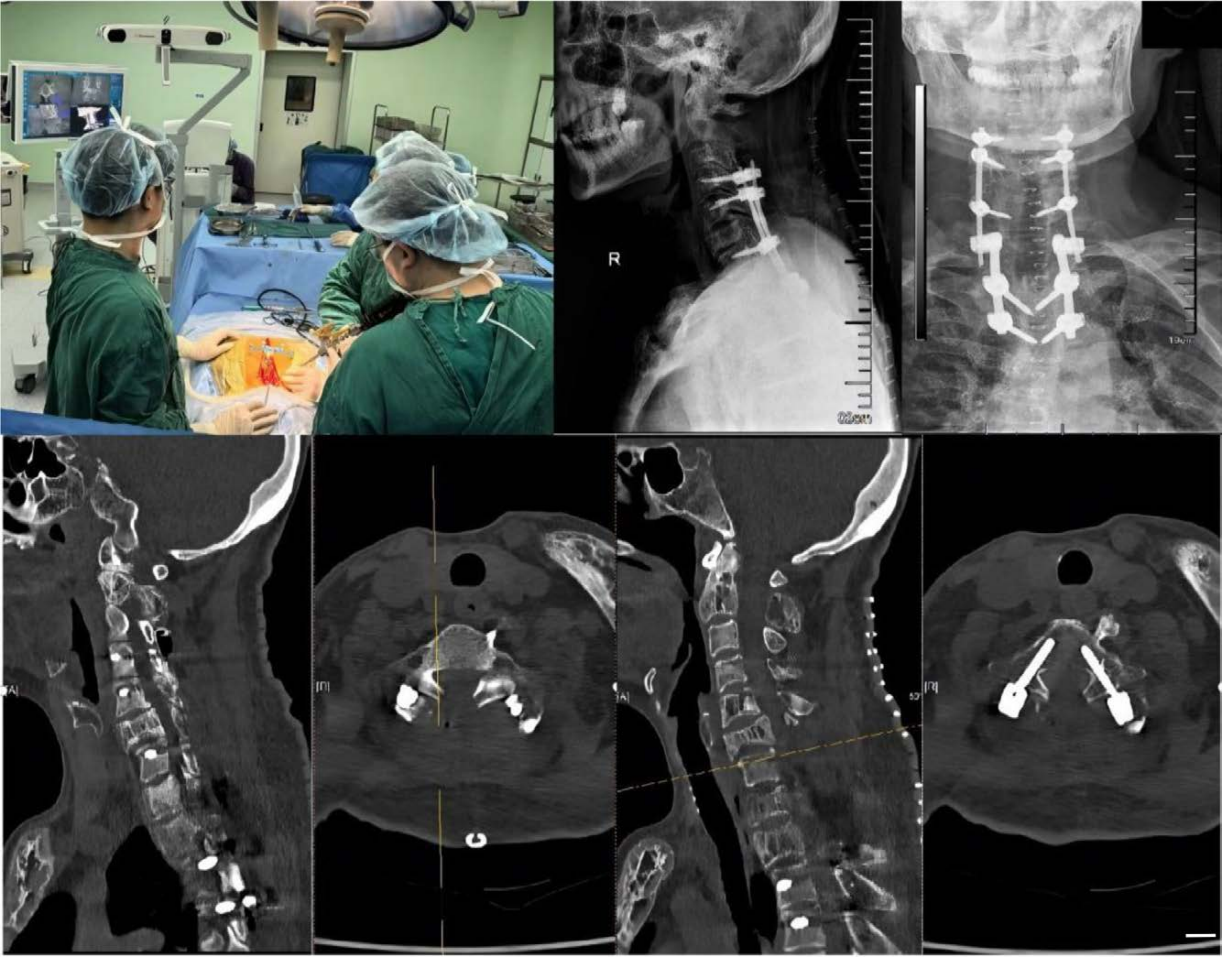
Postoperative imaging after internal fixation and decompression. X-ray and CT images confirm successful implantation of the internal fixation, which enhanced cervical stability and markedly reduced spinal canal stenosis and cord compression (scale bar = 2 cm). CT = computed tomography.

**Figure 3.**
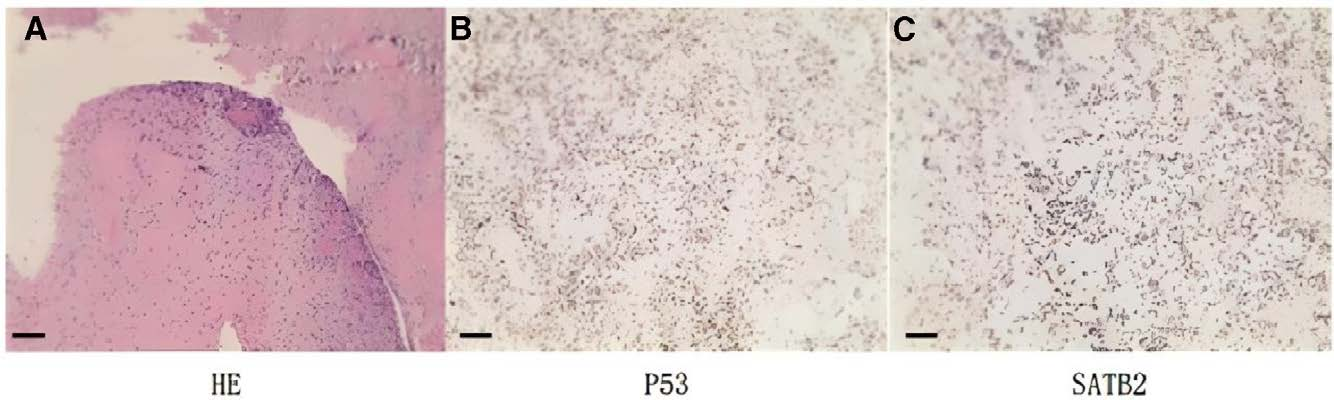
Intraoperative pathological and immunohistochemical findings of the T1 vertebra and appendages. The results of HE staining (A) showed that there were (T1 vertebral body) spindle cell malignant tumor with visible anisocytosis and bone-like stroma in some areas (scale bar = 100 μm); (B and C) The IHC staining showed the high possibility of osteosarcoma for the immunohistochemical results were as follows: P53 (diffuse +) (B), SATB2 (+) (C) (scale bar = 100 μm).

Further diagnostic workup confirmed thoracic spinal stenosis at the T2 level, and genetic testing identified dual SQSTM1 and ZNF687 mutations (Table 1), consistent with aggressive Paget disease. The test results for all mutated genes of this patient can be found in Table S1 (Supplemental Digital Content, https://links.lww.com/MD/R218). Intraoperative exploration identified destruction of the T1 vertebra and soft tissue compression; postoperative pathology confirmed malignancy (Fig. 2). Final histopathological examination confirmed osteosarcoma (spindle cell tumor positive for SATB2, Ki-67 50%, and P53) (Fig. 3).

**Table 1:** Information on variants found by genetic testing in this case report individual associated with Paget disease of bone.

Genetics	Chromosome location	Nucleotide variations	Amino acid changes	Pure/Hybrid	Normal population frequency	Pathogenicity analysis	inheritance	Gene function/disease phenotype
SQSTM1	5q53.3	c.1160C > T	p.P387L	Hybrid	–	Pathogenic	AD\AD\AD\AR	1.Paget disease of bone type 3; 2.Frontotemporal lobe dementia and/or amyotrophic lateral sclerosis type 3 3.Distal myopathy with marginal vacuoles; 4. Neurodegenerative disease with ataxia, dystonia and gaze palsy, childhood onset
ZNF687	1q21.3	C.2294 + 7C>T	–	Hybrid	0.0464	Uncertain	AD	Paget disease of bone type 6

### 2.3. Therapeutic intervention

The patient underwent surgical decompression via C6–T1 laminectomy. Short-term symptomatic improvement was observed postoperatively (reduced numbness and partial motor recovery). To inhibit tumor growth and osteoclast activity, denosumab (120 mg) was administered. Subsequent chemotherapy was considered based on the patient’s tolerance.

### 2.4. Outcomes

Despite treatment, the patient’s lower limb muscle strength declined to grade 1 by postoperative day 40. This case highlights the rarity of spinal malignant transformation in Paget disease, particularly in Asian populations, and underscores the importance of genetic profiling and long-term monitoring in high-risk cases. A detailed timeline summarizing key diagnostic, therapeutic, and outcome-related events is provided in Table 2 to clarify the disease course and management milestones.

**Table 2:** Chronological timeline of key events and changes.

Time point	Event type	Key results and changes
June 2019	Initial diagnosis	Diagnosed with Paget disease of bone (PDB) based on imaging findings (multifocal osteolytic destruction, cortical hyperplasia, and osteosclerosis) and elevated alkaline phosphatase (ALP) levels (>800 U/L). Initiated bisphosphonate therapy.
2019–2024	Follow-up and treatment	Annual outpatient monitoring with continued bisphosphonate administration; disease remained relatively stable without significant symptomatic deterioration.
October 25, 2024	Biochemical progression	ALP levels surged to 3100 U/L, indicating increased disease activity and potential malignant transformation.
November 14, 2024	Acute neurological event	The patient experienced sudden onset of numbness in both toes, which propagated to the chest within 24 h, accompanied by lower limb weakness (right limb muscle strength grade 4, left limb grade 3) and urinary/bowel dysfunction. Hospital admission was required for evaluation.
November 2024 (post-admission)	Diagnostic confirmation	Genetic sequencing revealed mutations in SQSTM1 and ZNF687 genes, confirming the hereditary basis of PDB. PET-CT showed increased glucose metabolism in multiple bones, suggesting osteosarcoma. Imaging identified thoracic spinal stenosis at the T2 level.
November 2024 (surgery)	Surgical intervention and pathological diagnosis	Laminectomy was performed; intraoperative rapid pathology indicated potential malignancy. Postoperative definitive pathology confirmed osteosarcoma (spindle cell malignant tumor with pleomorphism; immunohistochemistry: SATB2+, Ki-67 50%+, P53 diffuse+).
Postoperative day 3	Short-term outcome	Numbness alleviated without electric shock-like sensations; muscle strength improved (right limb to grade 4+, left limb showed partial improvement).
Postoperative day 14	Medium-term outcome	Left limb muscle strength recovered to grade 3, but thoracic and abdominal sensation showed no significant improvement.
Postoperative days 20–40	Long-term outcome	Muscle strength fluctuated with an overall decline (lower limb strength decreased to grade 1); no paradoxical increase in ALP levels was observed. Denosumab (120 mg) was administered to inhibit tumor growth, with chemotherapy considered based on patient tolerance.

PET-CT = positron emission tomography-computed tomography.

## 3. Discussion

This case report documents a rare instance of spinal PDB that transformed into osteosarcoma in a patient who presented to the hospital with a sudden onset of diplegia. Genetic testing revealed that the patient had mutations in both SQSTM1 and ZNF687.

Sequencing of bone metabolism-related genes in this patient revealed mutations in both the SQSTM1 and ZNF687 genes. Previous studies have shown that the pathogenesis and neoplastic transformation of Paget disease involve mutations in at least 2 genes (SQSTM1 and ZNF687).^[6,7]^ Current evidence suggests that the coexistence of SQSTM1 and ZNF687 double gene mutations may synergistically promote malignancy. In our clinical experience, this case underscores the potential aggressiveness of such double mutations, as the patient exhibited rapid biochemical progression (ALP surge to 3100 U/L) and early neurological complications, which may reflect a synergistic effect accelerating malignant transformation. Further validation of the direct role of ZNF687 in osteosarcoma development is required.^[6]^ ZNF687, which is highly expressed in osteoblasts and osteoclasts, may have a direct effect on the bone remodeling mechanisms of Paget disease^[6]^ and is more closely associated with the transformation of Paget disease into a giant cell tumor of bone.^[8,9]^ Recent studies have confirmed ZNF687 as the causative gene in patients with severe, early-onset, polyostotic PDB.^[10]^ Nevertheless, there is a paucity of research examining the correlation between ZNF687 gene mutations and the etiology of osteosarcoma.^[11]^ The latest research has shown that ZNF687 mutations exert distinct effects on osteoclastogenesis and skeletal remodeling. Specifically, the loss of Zfp687 results in the disruption of progenitor development and increases bone mass. In contrast, the P937R mutation leads to excessive bone resorption and pathological remodeling, characteristic of PDB.^[12]^ We observed that the patient’s ZNF687 mutation, combined with SQSTM1, may have exacerbated bone resorption and remodeling defects, contributing to the spinal stenosis and acute presentation – a pattern not thoroughly documented in Asian populations, highlighting the need for ethnicity-specific studies. Critically, impaired autophagy due to the SQSTM1 mutation may sensitize osteoclast precursors to ferroptosis, an iron-dependent cell death process recently implicated in bone homeostasis. The concomitant ZNF687-driven hyperactivation of osteoclasts could thus operate within a microenvironment primed for aberrant cell death, creating a vicious cycle that amplifies bone turnover and structural damage.^[13]^

As an autosomal dominant disorder, many mutations in SQSTM1 appear to be responsible for the predisposition to Paget disease in some families.^[14,15]^ Ten percent of patients present with familial PDB, and 35 percent of them carry mutations in the SQSTM1 gene. Mutations in the SQSTM1 gene mediate autophagy and thus cause abnormal bone metabolism.^[16]^ The majority of SQSTM1 gene mutations affect the ubiquitin-binding domain of the protein and are associated with more severe clinical manifestations of Paget disease of the bone.^[17,18]^ SQSTM1 mutations are the most common germline mutations in patients with disseminated and familial PDB.^[15,17]^ Compared with Western patients, Chinese patients have an earlier age of onset, more severe symptoms, and a lower frequency of SQSTM1 mutations, accounting for only 4.0% of cases.^[19]^ This suggests that Asian populations may possess a distinct genetic background or that environmental factors may modulate disease manifestations, warranting heightened clinical awareness. Recent studies have shown that testing individuals with a family history of PDB for pathogenic SQSTM1 gene mutations and performing radionuclide bone scans on those who test positive can be used to detect the disease at an early stage.^[20]^ In this case report, the inclusion of multiple genetic mutations beyond the canonical Paget disease-associated genes (e.g., LAMA5, ASPH, PLEKHG2) serves to provide a comprehensive genetic profile that supports holistic clinical interpretation and contributes foundational data for future research into novel genotype-phenotype associations in complex bone disorders.

To delve deeper into the genetic context and molecular mechanisms of this rare case, we considered recent evidence regarding hereditary susceptibility and transformative pathways. Accumulating evidence underscores the critical role of germline mutations in familial neoplastic disorders, potentially driving malignant transformation through synergistic signaling pathways. For instance, germline mutations in the *CHST15* gene (e.g., c.1367delG) have been associated with familial myeloproliferative neoplasms (MPNs). This mutation establishes a CHST15-JAK2-Stat3 signaling axis, enhancing cell proliferation and differentiation potential while activating an “inflammatory response-extracellular matrix-immune regulation” network, thereby increasing the risk of disease development and transformation.^[21]^ Similarly, the germline *KMT2A* G3131S mutation promotes cell proliferation, colony formation, and downregulates C-myb expression, reinforcing its role in genetically susceptible MPNs.^[22]^ Furthermore, the contribution of immune regulatory pathways to disease progression is significant. The interleukin-27 receptor alpha subunit (*IL27RA*), whose high expression in breast cancer is correlated with enhanced immune infiltration and improved response to immunotherapy, suggests its potential role in modulating the tumor microenvironment and malignant transformation.^[23]^ Collectively, these studies highlight the interplay between germline mutations and immune dysregulation in disease pathogenesis. In the present case, we hypothesize that the dual-gene co-mutation may drive the development and transformation of spinal Paget disease via analogous mechanisms, such as aberrant JAK-STAT signaling and immune evasion. Future studies are warranted to validate this hypothesis and uncover novel therapeutic targets.

There is a paucity of literature on spinal Paget disease sarcoma, particularly in East Asia where such cases are extremely rare and those involving mutations in genes such as SQSTM1 have not been reported in recent years.^[24,25]^ According to a study in the United Kingdom, the prognosis for patients with Paget disease sarcoma of the spine after surgical treatment or radiotherapy was <5 months on average.^[26]^ The acute neurological deterioration in this case highlights the management challenges posed by malignant transformation with spinal involvement, for which prognostic data are especially limited in Asian populations. Mutations in SQSTM1 may impair the antioxidant stress response mediated by the NFE2L2/Nrf2 pathway via selective autophagy^[27]^ and promote autophagy-associated ferroptosis.^[28]^ Such mutations are likely to cause damage to normal bone tissue, leading to disruption of the original bone microstructure. Meanwhile, ZNF687 is a transcription factor involved in osteoblast/osteoclast differentiation and promotes osteoclast activity.^[29]^ Therefore, under the influence of both SQSTM1 and ZNF687 mutations, increased damage to normal bone tissue coupled with enhanced osteoclast function may synergistically accelerate disease deterioration.

In the context of solid tumor management, prognostic biomarkers such as the Cachexia Index, neutrophil-to-eosinophil ratio, and Royal Marsden Hospital score are critically important for risk stratification and personalized treatment planning, as they help identify high-risk patients who may benefit from intensified systemic therapies.^[30–32]^ Spinal osteosarcoma, a challenging bone tumor subtype, primarily requires surgical resection, but systemic treatments become increasingly relevant in metastatic or high-risk scenarios.^[30–32]^ The advent of novel agents like immune checkpoint inhibitors and antibody-drug conjugates (ADCs) has transformed oncology care, though sex-based differences in immune checkpoint inhibitor toxicity, as highlighted in the MOUSEION-07 study, necessitate personalized management strategies.^[33]^ Datopotamab deruxtecan, a TROP-2-targeting ADC, has demonstrated efficacy in aggressive malignancies such as triple-negative breast cancer, prompting further investigation into its potential application in spinal osteosarcoma through antigen expression studies.^[34]^ Integrating these prognostic tools with advanced therapeutics enables tailored treatment approaches, improving outcomes in high-risk cases through multimodal care that combines surgery, systemic therapy, and biomarker-guided decisions.

Meanwhile, it is worth noting that although the median survival time for patients with Paget disease who develop spinal osteosarcoma is not high, adequate decompression surgery remains an effective means of relieving neurological or spinal cord compression symptoms and preventing further deterioration of neurological function. For patients with complete paraplegia caused by isolated spinal osteosarcoma, en bloc resection of the tumor combined with sacrifice of spinal cord contents can achieve long-term disease-free survival at the cost of sacrificing some function.^[35]^ In this case, the patient showed significant improvement in neurological function within one week postoperatively.

This study has several inherent limitations. Firstly, as a single-case report, the findings lack validation from multi-center studies and support from larger cohort samples. Secondly, the absence of biopsies from other skeletal sites (e.g., limb bones) may have compromised a comprehensive evaluation of malignant progression. Furthermore, although genetic data are extensive, the lack of functional validation and mechanistic exploration constrains a deeper mechanistic understanding of the synergistic effects of the double mutations.

To advance the understanding of the distinct characteristics and malignant transformation mechanisms of Paget disease in Asian populations, future studies should prioritize the establishment of a population-specific genetic database. Multi-center collaborations are recommended to integrate clinical and genetic data, with a focus on elucidating the molecular synergy of SQSTM1 and ZNF687 dual-gene co-mutations. Future studies employing mammalian models and cross-population genetic approaches are warranted to validate these findings and to elucidate the mechanisms underlying the notable ethnic disparities in disease susceptibility. Exploration of novel therapeutic approaches such as ADCs for spinal osteosarcoma should also be pursued, thereby facilitating the development of personalized treatment strategies.

## 4. Conclusion

This case aims to underscore the importance for physicians to consider the possibility of secondary malignancies during the follow-up of patients with Paget disease, particularly in the elderly demographic. Beyond standard blood biochemistry and imaging assessments, it may be prudent to recommend genetic testing for patients when feasible. The concurrent detection of elevated ALP levels alongside mutations in the SQSTM1 and ZNF687 genes should raise a strong suspicion of malignant transformation in Paget disease. In such instances, it is imperative to conduct a biopsy of the lesion promptly to ascertain its nature, thereby facilitating timely intervention.

## Acknowledgments

Sincere appreciation is given to all the authors who participated in writing, reviewing, revising and collecting the data and to the staff who made important contributions to this article.

## Author contributions

**Supervision:** Huilin Yang.

**Validation:** Linlin Zhang, Huilin Yang, Qin Zhang.

**Visualization:** Yu Wang, Linlin Zhang, Qin Zhang.

**Writing – original draft:** Yu Wang.

**Writing – review & editing:** Yu Wang.

## Supplementary Material



## References

[1] Appelman-Dijkstra NM Papapoulos SE. Paget’s disease of bone. Best Pract Res Clin Endocrinol Metab. 2018;32:657–68. 30449547 10.1016/j.beem.2018.05.005

[2] Cundy T. Paget’s disease of bone. Metabolism. 2018;80:5–14. 28780255 10.1016/j.metabol.2017.06.010

[3] Banaganapalli B Fallatah I Alsubhi F. Paget’s disease: a review of the epidemiology, etiology, genetics, and treatment. Front Genet. 2023;14:1131182. 37180975 10.3389/fgene.2023.1131182 PMC10169728

[4] Choi YJ Sohn YB Chung YS. Updates on Paget’s disease of bone. Endocrinol Metab (Seoul). 2022;37:732–43. 36327984 10.3803/EnM.2022.1575 PMC9633214

[5] Kravets I. Paget’s disease of bone: diagnosis and treatment. Am J Med. 2018;131:1298–303. 29752905 10.1016/j.amjmed.2018.04.028

[6] Russo S Scotto di Carlo F Maurizi A. A mutation in the ZNF687 gene that is responsible for the severe form of Paget’s disease of bone causes severely altered bone remodeling and promotes hepatocellular carcinoma onset in a knock-in mouse model. Bone Res. 2023;11:16. 36918542 10.1038/s41413-023-00250-3 PMC10014847

[7] Gennari L Rendina D Falchetti A Merlotti D. Paget’s disease of bone. Calcif Tissue Int. 2019;104:483–500. 30671590 10.1007/s00223-019-00522-3

[8] di Carlo FS Pazzaglia L Mumm S. ZNF687 mutations in an extended cohort of neoplastic transformations in Paget’s disease of bone: implications for clinical pathology. J Bone Miner Res. 2020;35:1974–80. 32106343 10.1002/jbmr.3993

[9] Divisato G Formicola D Esposito T. ZNF687 mutations in severe paget disease of bone associated with giant cell tumor. Am J Hum Genet. 2016;98:275–86. 26849110 10.1016/j.ajhg.2015.12.016 PMC4746367

[10] Huybrechts Y De Ridder R Steenackers E. Genetic screening of ZNF687 and PFN1 in a Paget’s disease of bone cohort indicates an important role for the nuclear localization signal of ZNF687. Calcif Tissue Int. 2023;113:552–7. 37728743 10.1007/s00223-023-01137-5

[11] Divisato G Scotto di Carlo F Petrillo N Esposito T Gianfrancesco F. ZNF687 mutations are frequently found in pagetic patients from South Italy: implication in the pathogenesis of Paget’s disease of bone. Clin Genet. 2018;93:1240–4. 29493781 10.1111/cge.13247

[12] Russo S Gianfrancesco F. OP0124 ZNF687 regulates osteoclastogenesis and drives Paget’s disease of bone through myeloid lineage populations. Ann Rheum Dis. 2025;84:110.

[13] Dixon SJ Olzmann JA. The cell biology of ferroptosis. Nat Rev Mol Cell Biol. 2024;25:424–42. 38366038 10.1038/s41580-024-00703-5 PMC12187608

[14] Rabjohns EM Hurst K Ghosh A Cuellar MC Rampersad RR Tarrant TK. Paget’s disease of bone: osteoimmunology and osteoclast pathology. Curr Allergy Asthma Rep. 2021;21:23. 33768371 10.1007/s11882-021-01001-2

[15] Singer FR. Paget’s disease of bone-genetic and environmental factors. Nat Rev Endocrinol. 2015;11:662–71. 26284446 10.1038/nrendo.2015.138

[16] Wang J Zhang Y Cao J. The role of autophagy in bone metabolism and clinical significance. Autophagy. 2023;19:2409–27. 36858962 10.1080/15548627.2023.2186112 PMC10392742

[17] Layfield R Hocking LJ. SQSTM1 and Paget’s disease of bone. Calcif Tissue Int. 2004;75:347–57. 15365659 10.1007/s00223-004-0041-0

[18] Gennari L Rendina D Merlotti D. Update on the pathogenesis and genetics of Paget’s disease of bone. Front Cell Dev Biol. 2022;10:932065. 36035996 10.3389/fcell.2022.932065 PMC9412102

[19] Tao X Liu L Yang X. Clinical characteristics and pathogenic gene identification in chinese patients with Paget’s disease of bone. Front Endocrinol (Lausanne). 2022;13:850462. 35355568 10.3389/fendo.2022.850462 PMC8959906

[20] Phillips J Subedi D Lewis SC. Randomised trial of genetic testing and targeted intervention to prevent the development and progression of Paget’s disease of bone. Ann Rheum Dis. 2024;83:529–36. 38123339 10.1136/ard-2023-224990 PMC10958267

[21] Chen Y Zhang Y Wang Z. CHST15 gene germline mutation is associated with the development of familial myeloproliferative neoplasms and higher transformation risk. Cell Death Dis. 2022;13:586. 35798703 10.1038/s41419-022-05035-w PMC9263130

[22] Yin L Xie S Chen Y. Novel germline mutation KMT2A G3131S confers genetic susceptibility to familial myeloproliferative neoplasms. Ann Hematol. 2229. 100:2229–40. 10.1007/s00277-021-04562-4 34228147

[23] Chen Y Anwar M Wang X Zhang B Ma B. Integrative transcriptomic and single-cell analysis reveals IL27RA as a key immune regulator and therapeutic indicator in breast cancer. Discov Oncol. 2025;16:977. 40450602 10.1007/s12672-025-02811-w PMC12127260

[24] Wat WZ Cheung WS Lau TW. A case series of Paget’s disease of bone in Chinese. Hong Kong Med J. 2013;19:242–8. 23568934 10.12809/hkmj133661

[25] Zhang Y Gao P Yan S. Clinical, biochemical, radiological, and genetic analyses of a patient with VCP gene variant-induced Paget’s disease of bone. Calcif Tissue Int. 2022;110:518–28. 34800131 10.1007/s00223-021-00929-x

[26] Arshi A Sharim J Park DY. Prognostic determinants and treatment outcomes analysis of osteosarcoma and Ewing sarcoma of the spine. Spine J. 2017;17:645–55. 27856382 10.1016/j.spinee.2016.11.002 PMC5561729

[27] Deng Z Lim J Wang Q. ALS-FTLD-linked mutations of SQSTM1/p62 disrupt selective autophagy and NFE2L2/NRF2 anti-oxidative stress pathway. Autophagy. 2020;16:917–31. 31362587 10.1080/15548627.2019.1644076 PMC7144840

[28] Yang L Ye F Liu J Klionsky DJ Tang D Kang R. Extracellular SQSTM1 exacerbates acute pancreatitis by activating autophagy-dependent ferroptosis. Autophagy. 2023;19:1733–44. 36426912 10.1080/15548627.2022.2152209 PMC10262765

[29] Varela D Varela T Conceição N Cancela ML. Epigenetic Regulation of ZNF687 by miR-142a-3p and DNA Methylation during osteoblast differentiation and mice bone development and aging. Int J Mol Sci. 2025;26:2069. 40076693 10.3390/ijms26052069 PMC11899743

[30] Bas O Sahin TK Karahan L Rizzo A Guven DC. Prognostic significance of the cachexia index (CXI) in patients with cancer: a systematic review and meta-analysis. Clin Nutr ESPEN. 2025;68:240–7. 40157535 10.1016/j.clnesp.2025.03.023

[31] Sahin TK Rizzo A Aksoy S Guven DC. Prognostic significance of the Royal Marsden Hospital (RMH) score in patients with cancer: a systematic review and meta-analysis. Cancers (Basel). 2024;16:1835. 38791914 10.3390/cancers16101835 PMC11120545

[32] Sahin TK Ayasun R Rizzo A Guven DC. Prognostic value of neutrophil-to-eosinophil ratio (NER) in cancer: a systematic review and meta-analysis. Cancers (Basel). 2024;16:3689. 39518127 10.3390/cancers16213689 PMC11545344

[33] Vitale E Rizzo A Maistrello L. Sex differences in adverse events among cancer patients receiving immune checkpoint inhibitors: the MOUSEION-07 systematic review and meta-analysis. 2024;Sci Rep. 14:28309. 39550353 10.1038/s41598-024-71746-z PMC11569249

[34] Schipilliti FM Drittone D Mazzuca F Forgia D La Guven DC Rizzo A. Datopotamab deruxtecan: a novel antibody drug conjugate for triple-negative breast cancer. Heliyon. 2024;10:e28385. 38560142 10.1016/j.heliyon.2024.e28385 PMC10981107

[35] Gaiani F Boriani S Gasbarrini A. En bloc resection, including the cord for tumor-free margin, in a multilevel osteosarcoma of the spine: 20-year disease-free survival. Illustrative case. J Neurosurg Case Lessons. 2025;9:1. 10.3171/CASE24771 PMC11894278 40064000

