# Supplementary material for: A 5-year Chinese longitudinal case report on the malignant transformation of spinal Paget disease
Source: Medicine (Baltimore). 2026 Jan 30;105(5):e47190. doi: 10.1097/MD.0000000000047190 (PMC12863807; doi:10.1097/MD.0000000000047190)
Supplement: Supplementary file 1 [file medi-105-e47190-s001.doc]

Supplementary materials

| **Genetics** | **Chromosome location** | **Nucleotide variations** | **Amino acid changes** | **Pure/**  **Hybrid** | **Normal population frequency** | **Pathogenicity analysis** | **inheritance** | **Gene Function/Disease Phenotype** |
| --- | --- | --- | --- | --- | --- | --- | --- | --- |
| SQSTM1 | 5q53.3 | c.1160C>T | p.P387L | Hybrid | - | Pathogenic | AD\AD\  AD\AR | 1. Paget's disease of bone type 3; 2. Frontotemporal lobe dementia and/or amyotrophic lateral sclerosis type 3 3. Distal myopathy with marginal vacuoles;   4. Neurodegenerative disease with ataxia, dystonia and gaze palsy, childhood onset |
| LAMA5 | 20q13.33 | c.10825G>T | p.A3609S | Hybrid | 0.011 | Uncertain | AR\AR | 1. Bent bone dysplasia syndrome type 2  2. Nephrotic syndrome type 26 |
| ASPH | 8q12.3 | c.703+1G>A | - | Hybrid | - | Likely pat  hogenic | AR | Traboulsi's syndrome |
| ZNF687 | 1q21.3 | C.2294+7C>T | - | Hybrid | 0.0464 | Uncertain | AD | Paget's disease of bone type 6 |
| PLEKHG2 | 19q13.2 | c.610C>T | p.R204W | Hybrid | 0.0015 | Pathogenic | AR | Cerebral white matter dystrophy and acquired microcephaly  With or without dystonia |
| NUP107 | 12q15 | c.431delA | p.D144fs | Hybrid | 0.0001 | Likely pathogenic | AR\AR\AR | 1. Ovarian hypoplasia type 6  2. Galloway-Mowat syndrome type 7  3. Nephrotic syndrome type 11 |
| ALDH4A1 | 1p36.13 | c.973delC | p.R325fs | Hybrid | - | Likely pathogenic | AR | Hyperprolinemia type II |
| CACNA1E | 1q25.3 | c.100G>A | p.G34R | Hybrid | - | Uncertain | AD | Developmental and epileptic encephalopathy type 69 |
| HFE | 6p22.2 | c.1897C>G | p.H63D | Hybrid | 0.0353 | Pathogenic | AR | Haemochromatosis type 1 |
| FLG | 1q21.3 | c.5717C>A | p.s1906X | Hybrid | 0.0134 | Pathogenic | AD\AR\- | 1. Ichthyosis vulgaris  2. Atopic dermatitis type 2, susceptible |
| ELMO2 | 20q13.12 | c.1416+41C>T | - | Hybrid | 0.0031 | Uncertain | AR | Primary intraosseous vascular malformation |

**Table S1.** Information on all variants found by genetic testing in this case report individual.
